# The Registration of Nursing Homes

**Published:** 1907-11-30

**Authors:** 


### Nursing Administration.

### THE REGISTRATION OF NURSING HOMES.

### III. A QUESTION FOR THE NURSING PROFESSION.

The evils which are inflicted on the nursing profession by the existence of numerous nursing homes unregulated by law, and imbued with no sense of responsibility towards the public, have never yet been fully appreciated. There have not, however, been wanting those who have seen in these establishments the germ of all that want of discipline in the nursing world deplored by nurses and the public alike. Miss Monk, writing in the *Monthly Review in* 1905, maintained that the cure for the evils rampant among nurses was not registration of nurses, but the registration of homes, and not merely of those institutes which despatch nurses to the public, but of those establishments which undertake the nursing of private patients. The Royal British Nurses' Association long ago tacked on to their proposal for registration of nurses a measure for dealing with nursing homes, and in general it may be said that all who have studied the question are aware that no proposal for regulating the nursing profession can achieve its aims unless this breeding ground, for so it may be called, of spurious nurses is dealt with simultaneously. So acute is this aspect of the question, however, that it would seem futile to link to it reforms of a far-reaching and doubtful character. Having detected the source from which the spurious nurse is foisted on the public, would it not be better to let less pressing matters wait, and concentrate entirely on the best methods of counteracting a poison which is contaminating the nursing service throughout the kingdom?

It may be safely asserted that the only possible way in which untrained nurses can get the opportunity of being introduced into a private practice is through the medium of a nursing home. This is so well known that large nursing homes, managed on commercial principles, are able to insure a continuous flow of "probationers" who will pay high premiums for the privilege of an introduction to the medical men frequenting the home. In garments of the most austere hospital cut, in snowy caps, with an air of deepest submission, and, it must be conceded, with considerable natural aptitude, these women pose under the eye of the doctor as admirable nurses. Their ignorance is so artless, their desire to learn so obvious—they take so adroitly, in fact, the measure of those with whom they have to do—that all deficiencies are condoned, and a reputation is established as an accommodating nurse. From this to obtaining private work under the doctors to whose notice she has thus been brought is but a step. The "probationer" leaves the home after a year or so of miscellaneous attendance on people who believe her to be a trained nurse, and blossoms out as a skilled article under the good-natured recommendation of doctors she has studied to please. It is, of course, not the want of knowledge which is the most deplorable feature in a nurse introduced to responsible duties in this hole-and-corner fashion. She has absolutely no sense of the honour of her calling. She has none of the traditions of the training school to guide her. She has a smattering of nursing lore which makes her dogmatic to her patients, and often most disloyal to those under whom she works. And, alas! she cares in the last resort very little indeed about nursing at all. She has chosen her walk in life with a view purely to self-advancement, and in advancing what she believes to be her own interests she will not be particularly scrupulous. It is imperative in the best interests of nurses that these short-cuts to the practice of nursing should be effectually blocked. The presence of the "probationer" in the nursing home is bad for her, and from the point of view of the patient absolutely indefensible. The hospital probationer works under the immediate eye of her superiors. The sister, staff nurse and senior probationer are witness of her proceedings, and only the most elementary assistance in the wards is rendered by her until she has regularly learnt her duties. But this training and supervision are impossible among patients nursed in separate rooms. Under such conditions the blunders of the raw hand may be irreparable, and the discomfort she causes can never be brought to light. The one advantage which it may be thought that the private patient is entitled to is the absence of the unskilled nurse. In a properly managed nursing home a fully-trained nurse is assigned to every acute case, and in no case is any office performed for a patient by any other than a qualified woman.

But the superintendent of the ordinary commercial nursing home is not necessarily herself a very competent nurse. She has invested her savings in the business, without any natural aptitude for it, with golden dreams of big profits, and with but the smallest conception of the heavy expenses entailed. She is speedily disillusioned, and the only method of saving which appears to be within her reach is to keep under the cost of the nursing. She can get a smart-looking, fever-trained or cottage-hospital nurse eager for further "experience" at a low figure, and in this way the "probationer" is supplemented and the home staffed. It should be remembered that most people make their first and last experience of "hospital nurses" in the walls of some nursing home to which they have been consigned for recovery after operation, or for some long-drawn-out rest cure. To hear such people, often kindly and fair-minded, inveighing against nurses all the rest of their lives on the strength of the discomforts endured through the cheap and nasty nursing of the nursing home is to realise how fatally easy it is to start a prejudice, and how impossible to remove it. In their own interests, for the credit of nursing as a whole, all superintendents of nursing should unanimously join in the movement which seeks to place nursing homes under inspection, and restrain them from deluding their patients with untrained women introduced by this means to opportunities for doing even more serious mischief among the public.

